# Three-dimensional chromatin in infectious disease—A role for gene regulation and pathogenicity?

**DOI:** 10.1371/journal.ppat.1009207

**Published:** 2021-02-04

**Authors:** Sage Z. Davis, Thomas Hollin, Todd Lenz, Karine G. Le Roch

**Affiliations:** Department of Molecular, Cell and Systems Biology (MCSB), University of California Riverside, California, United States of America; Joan and Sanford I Weill Medical College of Cornell University, UNITED STATES

## Abstract

The recent Coronavirus Disease 2019 pandemic has once again reminded us the importance of understanding infectious diseases. One important but understudied area in infectious disease research is the role of nuclear architecture or the physical arrangement of the genome in the nucleus in controlling gene regulation and pathogenicity. Recent advances in research methods, such as Genome-wide chromosome conformation capture using high-throughput sequencing (Hi-C), have allowed for easier analysis of nuclear architecture and chromosomal reorganization in both the infectious disease agents themselves as well as in their host cells. This review will discuss broadly on what is known about nuclear architecture in infectious disease, with an emphasis on chromosomal reorganization, and briefly discuss what steps are required next in the field.

## Author summary

In this review, we examine the current state of nuclear architecture in infectious diseases with an emphasis on chromosomal reorganization. Nuclear architecture plays an important role in regulation of transcription for several pathogens, as well as inflammatory responses in their host. Recent advances in technologies such as Hi-C have allowed in-depth studies of chromosomal reorganization during infectious disease development and provided insights into transcription mechanisms and pathogenicity. In addition, it has been demonstrated that pathogens can also affect/utilize the hosts nuclear architecture. These areas are heavily understudied in pathogens, and we hope this review will provide a comprehensive review on the current state of nuclear architecture in infectious diseases and provide an additional avenue for eradication efforts.

## Introduction

The recent Coronavirus Disease 2019 (COVID-19) pandemic, as of November 2020, has claimed over 1,200,000 lives and infected 50.7 million people globally, renewing urgency for studying and understanding the molecular mechanisms driving infectious disease in general [1]. Although great progress has been made about our understanding of the pathogens responsible for these diseases, one area that is still understudied today is how these infectious agents can tightly control their transcription, evade their host immune system and more specifically for the purpose of this review, what is the role of nuclear architecture in the development of these pathogens and their host cells. Nuclear architecture, or the physical arrangement of the genome and its components in the nucleus (section 1) [2], is a process known to play an essential role in the regulation of gene expression, DNA replication, DNA repair, RNA processing, and mRNA transport to name a few [3]. Nuclear architecture can range anywhere from whole chromosomal level (e.g., chromosome organization) to individual gene level (e.g., gene accessibility). Recent work from model organisms have highlighted the importance of nuclear architecture in many biological processes, thanks in large part to recent technologies such as Genome-wide chromosome conformation capture or Hi-C (section 2), which allow for the global snapshot of chromosome organization within cells, revealing proximity of functional elements from one another [4].

Deciphering the role of nuclear architecture in infectious agents, as well as the impact they can have on the nuclear architecture of their host cells, is fundamental to understanding the biology of these pathogens. Although there are many articles discussing the impact of local nuclear architecture in disease progression (e.g., Histone methylation, acetylation, DNA methylation, DNA accessibility, etc. (see section 1)), there is only a handful of scientific literature regarding chromosome level reorganization with regard to infectious disease agents. With recent adaptations of technology in infectious diseases, it is now possible to effectively study chromosomal reorganization, the process in which organisms alter their chromosomal configuration, throughout pathogens development in a high-throughput manner (see section 2) [5]. Recent studies in several organisms have investigated the changing chromosome organization during disease progression and have highlighted the importance of chromatin structure for the overall biology of infectious disease agents [6–8]. It is now known that like many other organisms, eukaryotic pathogens such as protozoan parasites can undergo drastic changes in chromosomal organization during their development. Those changes include alteration of their chromatin states [euchromatin and heterochromatin], histone modifications, genome accessibility, long noncoding RNA (lncRNA) expression/function, and many other factors [6,7,9]. Recent reports have also showed that virus infection can alter the host nuclear architecture, further highlighting the importance of understanding the effects of nuclear architecture in infectious diseases in general [10,11]. Ideally, with enough understanding of the regulation of chromatin structure, both in infectious disease agents and their host cells, we can target vulnerabilities in the alteration of the nuclear organization that can be further exploited to suppress diseases progression.

This review will provide a conspectus on the state of nuclear architecture today in infectious diseases with a heavy focus on chromosome reorganization. Due to the amount of literature available, heavy emphasis will be placed on *Plasmodium* species, that cause malaria in human. Recent findings about *Toxoplasma*, *Trypanosoma*, *Trichomonas vaginalis*, and other pathogens and how their nuclear architecture may play a role in pathogenicity will be covered. We will also assess how viral infections can affect the host nuclear architecture and discuss their implications in disease development. Finally, we will review the current knowledge gaps and discuss how an improve understanding of the role of nuclear architecture in infectious disease may help the eradication of some of these pathogens.

### Quick review of mechanisms affecting nuclear architecture

**Fig 1:**
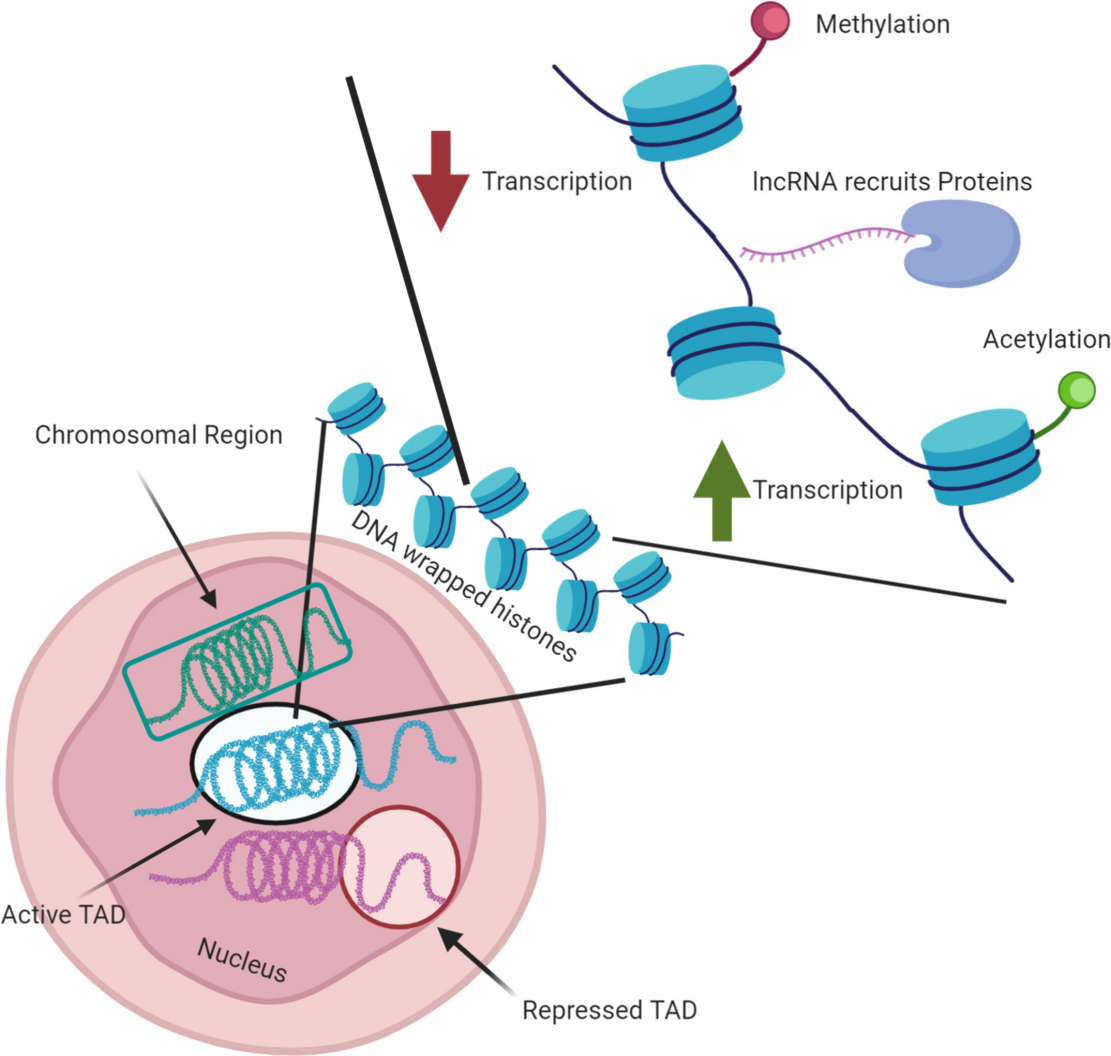
Highlighting the major factors in nuclear architecture. Although many processes are involved in nuclear architecture, we highlight here some of the major factors, ranging from chromosome [TADs, Chromosomal region] to gene [Histone acetylation, histone methylation, lncRNA recruiting proteins] organization. It should be noted here that much of the knowledge regarding nuclear architecture organization has been learned from complex eukaryotic cells, and these processes may or may not be present in all infectious disease agents. Figure created with biorender.com. lncRNA, long noncoding RNA; TAD, topologically associating domain.

This last decade, the study of the eukaryotic nuclear organization has at least partially revised our vision of gene regulation. It is now clear that structure of the chromatin is highly dependent on various conditions such as cellular environment, cell cycle, or cell type, which allow an adaption of its gene expression. Chromatin is a complex of DNA and proteins, which forms chromosomes within the nucleus of eukaryotic cells. It allows the several meters of DNA to be compacted and wrapped around a histone octamer to form nucleosomes, themselves packaged together in chromatin fibers. When the chromatin is highly condensed, the transcriptional machinery necessary to activate transcription is strongly hampered similarly to other protein complexes involved in diverse biological processes. A reorganization of the chromatin and the nucleosome is then necessary to ensure accessibility and transcription of the targeted genes. Modifications of the histone core, either by the presence of histone variants or by histone posttranslation modifications (PTMs), play a key role in chromatin organization. The distribution of the classical histone octamer, composed of 2 copies of H2A, H2B, H3, and H4, can vary, and several histone variants have also been identified as critical in chromatin reorganizations. One of them, the histone variant H2A.Z that was first identified in *Tetrahymena thermophila* in transcriptionally active macronucleus [12], has also been identified at the transcription start site of active and inactive genes [13–17] or at the enhancers [17,18]. In yeast, the C-terminal region of H2A.Z has been demonstrated to interact with RNA polymerase II (RNAPII), promoting its recruitment at active promoters [19]. It has also been implicated in heterochromatin regulation [20] and antisilencing function [21]. The protein also seems to facilitate DNA replication [22,23]. PTMs such as acetylation or methylation of histone N-terminal tails provide an additional level of regulation. Acetylation of histone H3, H3K9ac, and H3K14ac is generally associated with open chromatin and transcription [24,25], while the trimethylation H3K9me3 and H3K27me3 promote transcriptional repression [25,26]. Several other histone variants and PTMs have been reported to contribute to the regulation of gene expression (Fig 1) [27,28].

In addition to compaction, chromatin looping enables to counter the spatial distance that may exist between promoters and *cis*-regulatory DNA elements. As examples, this mechanism has been reported with high-level expression of the β-globin gene cluster by the locus control region (LCR) distributed 30 to 60 kb upstream of the *β*^maj^*globin* gene [29–35], or silencing in the regulation of Polycomb genes in Drosophila [35–39]. Recently, considerable attention was turned on the role of lncRNAs on chromatin structure. While some of their biological function remain obscure, there is little doubt that lncRNAs are key component of gene regulation and chromatin structure. Several lncRNAs have been shown to affect chromatin structure, histone marks, and transcription in cis and trans, often by recruiting protein complexes to specific genetic regions [40–43]. At a megabase scale, chromosomal regions are arranged in topologically associating domains (TADs) with high levels of chromatin loops detected to adjacent loci [44–47]. TAD boundaries are enriched in insulator binding protein CTCF [44,47] and histones modifications like H3K4me3 and H3K36me3 [44]. Furthermore, the chromosomes are also not randomly arranged in the nucleus; they gather in distinct regions, known as chromosomes territories, according to the state of chromatin condensation and organization [48–58].

### Quick review of technologies used to study nuclear architecture

Given that spatial organization of chromatin within the nucleus and interactions between loci at 3-dimensional level play a central role in gene expression, high-throughput methods to analyze these physical properties have been developed [59,60]. The original chromosome conformation capture (3C) technique, although elegant in its design, was limited because it only allowed the analysis of singular pairs of interacting loci, missing de novo identification of interaction sites (e.g., promoter–enhancer interactions) [61]. Using the same basic principle of proximity ligation and enzymatic digestion, further development of 3C-based techniques together with microarrays, described as chromosome conformation capture-on-chip (4C), allowed for an unbiased genome-wide search for DNA loci that interact with a given locus in the nuclear space [62,63]. Parallel development of the chromosome conformation capture carbon copy (5C) procedure aided in finding all interactions within a particular genomic domain [64,65]. Further developments, such as Chromatin interaction analysis by paired end tagging (ChIA-PET) combines 3C with chromatin immunoprecipitation (ChIP), have further improved chromosome capture techniques by providing an unbiased view of chromosomal interactions [66].

**Fig 2:**
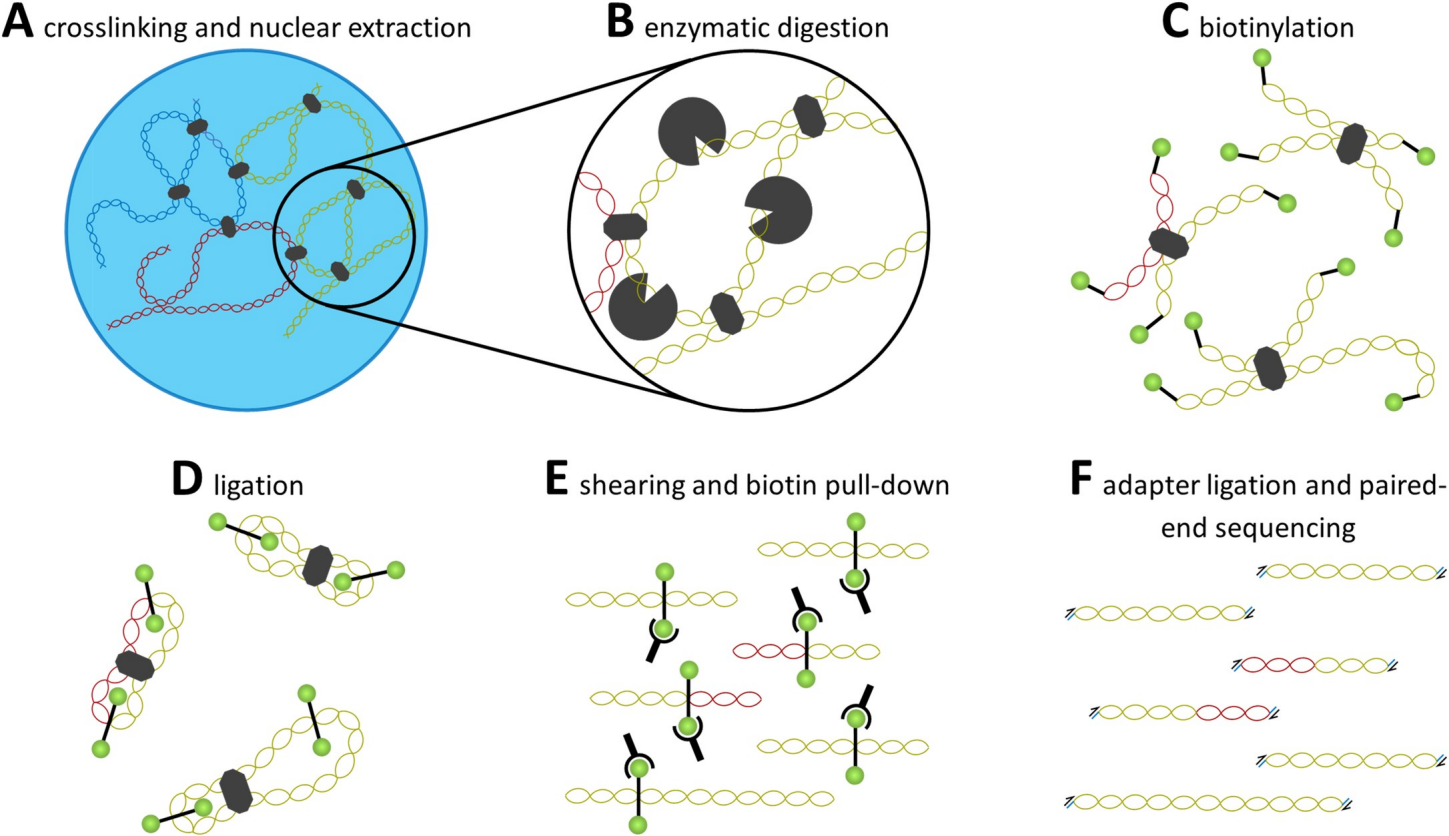
Overview of Hi-C procedure. A quick overview of the Hi-C chromatin capture procedure is described here. Proteins are cross linked with DNA, holding together sections of the chromatin that are in close proximity to one another. Enzymatic digestion and biotinylation separate genetic segments and tag them for later pull down. Ligation then connects the pieces of DNA near one another, which allows for hybrid DNA to be sheared, pulled down, and prepared for sequencing.

Rather than constrain analyses to predetermined loci, a derivative of 3C coupled with massively parallel next-generation sequencing was developed to quantify chromatin interactions on a genome-wide scale called Hi-C (Fig 2) [5,49]. Initial steps involve crosslinking chromatin by subjecting nucleated cells to formaldehyde fixation. To isolate crosslinked fragments, the cells are lysed and chromatin within the nucleus is digested using one or more restriction enzymes. The restriction enzyme used for enzymatic digestion is chosen based on the desired frequency of endonuclease recognition sequences. A 4-bp cutter such as MboI (GATC) will, in principle, cut the chromatin at a higher frequency than a 6-bp cutter such as HindIII (AAGCTT) [6,44,49,67,68]. It is possible to use multiple endonucleases simultaneously to increase the number of cut sites, consequently resulting in breaks closer to the crosslinked sequence, thus increasing the resolution of the sequence [69]. However, the proposed frequency of cut sites for each restriction enzyme is based on the frequency of the 4 nucleotides in a hypothetical “perfect” genome, such that the frequency of AT and GC pairs is both equal and evenly distributed, and the number and distribution of cut sites will differ between organisms and cell types. Following digestion, the 5′ overhang on each fragment is filled in using a biotinylated residue, and the blunt ends are ligated together. The DNA is then sheared and decrosslinked, then the fragments containing biotinylated residues are collected. Adapters are ligated to the fragments and they are analyzed using paired-end sequencing platforms.

### Chromosome reorganization in eukaryotic pathogens

#### Plasmodium falciparum

**Fig 3:**
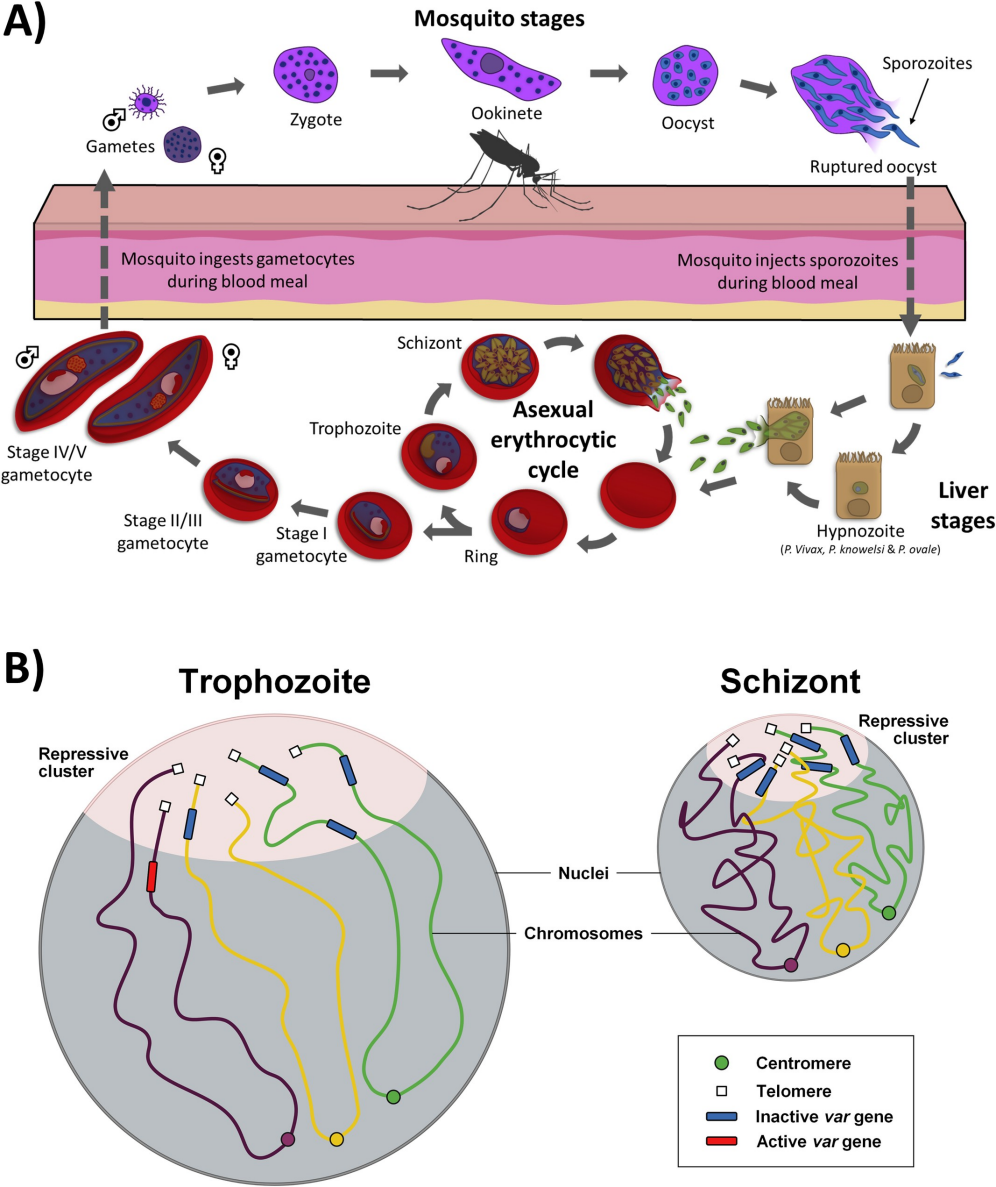
Life cycle of *Plasmodium* species and visual representations of VRSM genes in *P*. *falciparum*. [A] The life cycle of the *Plasmodium* species through its 2 hosts, mosquito and human stages. Note that in *P*. *vivax*, *P*. *ovale*, and *P*. *knowlesi*, the parasite can exhibit dormant hypnozoites stage in the liver. [B] The nuclear organization of 2 representative stages [trophozoite and schizont] of *P*. *falciparum* is shown with euchromatin and heterochromatin form, respectively. At the trophozoite stage, open chromatin structure allows for intensive transcription, while the majority of *var* genes that cluster together are repressed. During the schizont stage, more compact chromatin structure decreases overall transcription with the exception of gene involved in red blood cell invasion. For both stages, centromeres and telomeres tend to cluster with each other respectively. Figure created with biorender.com.

Much of our knowledge about nuclear architecture in pathogens comes from *Plasmodium* species such as *P*. *falciparum*, a causative agent of human malaria. *P*. *falciparum* accounts for more than 90% of the world’s malaria mortality, infecting an estimated 24 million children, of which an estimated 1.8 million were thought to have severe anemia in 2018 [70]. Recent reports of *P*. *falciparum*’s resistance against artemisinin and derivatives in Asia raised serious concerns about the future of malaria eradication [71–73]. All 5 *Plasmodium* species infecting human have similar genome sizes consisting of 14 chromosomes of approximately 22 to 33 million bases. They also have similar life cycles that involve 2 major hosts: the *Anopheles* mosquito and the human host. For *P*. *falciparum*, the human infection starts as a female mosquito takes a blood meal and injects sporozoites into the host bloodstream. In the host, the sporozoites travel into the liver to invade hepatocytes. After intensive differentiation and replication process, thousands of newly formed merozoites are released from the liver into the blood stream to invade red blood cells where they begin a 48-hour asexual replication cycle (Fig 3A) [74]. During the intraerythrocytic developmental cycle (IDC), the parasite progresses through 3 distinct stages termed ring, trophozoite, and schizont, and multiplies into 16 to 32 daughter cells that will burst out and invade new healthy erythrocytes. During the IDC, environment and stress can trigger sexual differentiation of parasites into male and female gametocytes. When mature, gametocytes ingested by a feeding mosquito can undergo sexual replication in the mosquito midgut. They further differentiate and multiply, then invade the salivary gland, where sporozoites can then be transmitted to a new human host. Although all parasitic stages have been investigated for therapeutic intervention, heavy emphasis has been placed on the erythrocytic stages, as these cause many symptoms associated with the disease (Fig 3A). A combination of advanced microscopy methodologies such as electron microscopy and Fluorescence in situ hybridization (FISH) as well as Hi-C experiments, which aid in constructing a 3D model of the nucleus at distinct stages of development cycle, have demonstrated that centromeres and telomeres cluster in opposite regions of the nucleus and that chromosomes are arranged into folded structures anchored at the centromere with both chromosome arms folding over parallel to each other [6,7]. Most importantly, such experiments have unveiled that parasite nuclear architecture undergoes drastic changes during its life cycle. These changes are most likely refining transcriptional activity taking place during the parasite developmental program. As an example, most of the active transcriptions during the IDC occur during the trophozoite stage, a stage similar to G1 phase in eukaryotic cell division, where the malaria parasite begins the production of RNAs and proteins to prepare for replication [75,76]. At that stage, most of the genome is open to a euchromatin state, consistent with the heavy active transcription with the exception of genes involved in virulence factors or sexual differentiation [6]. At the ring and schizont stages corresponding to G0 and S phases of the IDC respectively, the genome is relatively closed into a more heterochromatin form with the exception of genes involved in virulence factors such as genes involved in red blood cell remodeling or invasion of the host cell respectively. Furthermore, genes that colocalize together tend to be activated or repressed in conjunction with genes in close proximity, further evidencing that genome architecture can play a crucial role in transcriptional regulation of malaria parasites.

The strongest evidence we currently have on the importance of genome architecture in transcriptional regulation stems from the virulence genes known as VRSM genes (*var*, *rifin*, *stevor*, and *Pfmc-2tm* genes) (Fig 3B) [6]. VRSM proteins are encoded by large polymorphic gene families that are expressed in a mutually exclusive manner and are imperative to the parasite’s ability to evade the host’s immune response, a process similar to the “allelic exclusion” described for mammalian immunoglobulin (Ig) and odorant receptor genes [6,77]. Deciphering the principles that govern mutually exclusive expression of VRSM genes in *Plasmodium* has been a key question in the field. Previous studies have shown local factors that may play a role in regulation of virulence genes, such as histone methylation and suppressors [78–80]. Initial FISH experiments determined that some of the VRSM genes colocalize at the periphery of the nucleus [81]. Recent Hi-C experiments demonstrate that chromatin looping brings all VRSM genes including internal *var* as well as repressed genes physically close to each other near subtelomeric chromosomal regions [6]. The clustering of the *SICAvar* genes, virulence genes in *Plasmodium knowlesi*, a malaria parasite infecting *Macaca fascicularis* but increasingly recognized as a significant cause of human malaria, was also observed using Hi-C experiment, suggesting that the control of mutually exclusive gene expression of malaria virulence genes may be controlled at least partially at the nuclear architecture level [7]. It should be mentioned, however, that these results arising from Hi-C and FISH experiments are only corollary, and further research will be required to validate these findings, especially regarding the specific mechanisms behind control of virulence gene expression, and their importance for infectious disease research.

Another piece of evidence that suggests the importance of nuclear architecture in malaria is the organization of ribosomal DNA (rDNA) in *P*. *falciparum* [6]. rDNAs in *P*. *falciparum* are dispersed on different chromosomes and are known to be strictly regulated in the lifecycle. A previous FISH study suggested that all rDNA units localize at a single nucleolus with 2 units on chromosomes 5 and 7 that are actively transcribed during the IDC (A-type units) [82]. Hi-C data confirmed overall enrichment of contacts between chromosomes 5 and 7 in 3D models for the ring stage and place these 2 A-type rDNA units near the nuclear periphery suggesting the existence of perinuclear transcriptionally active compartments [6]. Such compartments may play a role in separating out the single active *var* gene per cell from compact chromatin around subtelomeric regions. No clustering or contact of the rDNAs (S-type units) transcribed in the mosquito’s vector were detected. This observation suggests that genomic location may influence rDNA expression by the preferential colocalization of active unit and that genome architecture plays a crucial role in the transcriptional regulation in malaria.

More recent studies have also started exploring the genome architecture of the transmission stages of *Plasmodium*, where the parasites transition to and from the human host to the mosquito host. Understanding the molecular mechanisms that control transition stage from human to mosquito hosts, known as gametocytes, and mosquitoes to human known as sporozoites is crucial to the eradication program of malaria around the world; however, little was known about the role of nuclear architecture in controlling these stages. Bunnik and colleagues used Hi-C on gametocyte and sporozoite stages in 2 human malaria parasites, *P*. *falciparum* and *P*. *vivax* to understand the genomic architectural changes that occur in the parasite during these stages [7]. During the gametocyte stages, the nuclear architecture of *P*. *falciparum* has similarities but strong particularities when compared to the IDC. Overall features such as the clustering of centromeres, telomeres, and virulence genes are consistent with the IDC; however, closer inspection reveals specific intrachromosomal reorganization. One key difference observed in gametocyte is the dissociation of the gametocyte-specific transcription factor *pfap2-g* (PF3D7_1222600) from the repressive center [7]. During the IDC, *pfap2-g* clusters with the virulence genes that are heavily repressed during the intraerythrocytic stages. During or shortly prior to early gametocyte stages, the *pfap2-g* gene disassociates from this virulence cluster, most probably required for the transcription of this master regulator of gametocytogenesis [7]. At the late gametocyte stage, *pfap2-g* was localized back to the repressive cluster. This is consistent with previous research showing that the expression levels of PfAP2-G highly correlate with gametocyte production, illuminating the correlation of nuclear architecture and the induction of gametocytes, and possibly, for the transmission of malaria between hosts. In sporozoites, the study highlighted differing contacts for genes involved in hepatocyte invasion in *P*. *vivax*. In *P*. *falciparum*, a strong reorganizing of rDNA repressed in the mosquito’s stage was also observed [7]. Although these results show that nuclear architecture may once again be playing a role in differing stage progression, further research is required to determine the extent and importance of nuclear architecture in the sporozoites.

Nuclear architecture studies have been highly useful to visualize changes in the genome organization, and to confirm these changes may play a significant role in the regulation of transcription. However, nuclear architecture studies can also be used to discover functions of genes previously unknown. One example of this is the transcription factor PfAP2-O3 (Pf3D7_1429200). Although the function of PfAP2-O3 as a transcription factor was previously known, it was unclear what genes were targeted and what role it played in the biology of malaria parasites [83]. However, Bunnik and colleagues found that during sexual differentiation, chromosome 14 displayed 2 superdomain-like structures, similar to what was observed in the inactivated X chromosome in humans and other mammals [84,85]. The boundary of these 2 domains was found relatively close to *pfap2-O3*, and through IFA studies, Bunnik and colleagues showed that this transcription factor was only active in female gametocytes and zygotes, leading to the discovery that this transcription factor may play a crucial role in sex determination [83].

### Nuclear architecture of other pathogens

Although most of the research of nuclear architecture in eukaryotic pathogens has focused on *Plasmodium* species, recent research has revealed new insights in nuclear architecture in other infectious disease agents. Two such examples are *Toxoplasma gondii* and *Babesia microti*, 2 apicomplexan parasites that infect humans. *T*. *gondii*, the causative agent of toxoplasmosis, is one of the world’s most common parasites. Infection usually occurs by eating contaminated meat, exposure from infected cat feces, or mother-to-child transmission during pregnancy. While most infected people have little to no symptoms, women newly infected with *Toxoplasma* during pregnancy or people with a compromised immune system can have severe symptoms. *B*. *microti* is usually transmitted by tick and is the causative agent of babesiosis. While rare, it can lead to life-threatening infection similar to malaria. Unlike in human *Plasmodium* species, Bunnik and colleagues showed that the genome organization in both *B*. *microti* and *T*. gondii at different stages of their life cycles both showed little correlation between chromosome organization and control of transcription [7]. Although *B*. *microti* shows some colocalization of virulence genes, the authors were unable to detect any significant correlated with gene expression. *T*. *gondii*, on the other hand, showed little to no colocalizations of virulence genes, but an increase gradient of gene expression detected from the centromere to the telomeres, suggesting *T*. *gondii* may utilize different modes to control virulence gene expression while the nuclear periphery may function to silence certain genes [7]. One possibility for the difference observed between *Plasmodium* species and *T*. *gondii* may be due to the large difference in known transcription factors available in their genomes. While 67 AP2 transcription factors were detected in the *Toxoplasma* genome, only 27 have been detected in *P*. *falciparum*, suggesting that in *T*. *gondii*, chromosome reorganization may play a lesser active role in transcription regulation [7]. Another possibility is the recently discovered MORC proteins, a set of proteins that create DNA loop entrapments that act as repressors [86,87]. Improved experimental approaches may be needed to increase the resolution of 3C techniques to detect chromatin remodeling events induce by MORC proteins in *T*. *gondii*. These results, however, raise interesting questions about the evolutionary differences and pathogenicity that lead to drastic altered transcriptional regulation between these related parasite species.

Another eukaryotic pathogen where the role of genome organization has been intensively studied is *Trypanosoma brucei*, a parasitic kinetoplastid transmitted by the tsetse fly in sub-Saharan Africa and causing African trypanosomiasis or sleeping sickness. The disease can be fatal if trypanosomes enter the brain causing a range of neurological symptoms including disturbance of sleep patterns. Although infection with *T*. *brucei* is in decline, the illness remains a major cause of poverty in sub-Saharan Africa. Similar to *var* genes in *P*. *falciparum*, *T*. *brucei* encode an array of variant surface glycoproteins or VSG-gene involved in antigenic variation, many in close proximity of the telomere ends of its many chromosomes [88]. Tight, mutually exclusive control of antigenic variation at the surface of the parasite enables the expression of one of these genes at a time allowing the parasite to evade the host immune system and persist in the host for decades [89]. Here again, Hi-C experiment suggests a distinct partitioning of the genome, with antigen-encoding subtelomeric regions folded into distinct, highly compact compartments at the nuclear periphery characteristic of silenced chromatin probably to silent most VSG genes [88]. In addition to colocalization at the chromatin structure level, Muller and colleagues found that 2 histone variants called H3.V and H4.V have a role in this expression-switching process [88]. Experiments using RNA-seq and Hi-C indicated that the absence of H3.V, but not of H4.V, nuclear organization is altered and resulted in increased clustering of telomeres at the nuclear periphery, but no change in gene expression was observed. When both H3.V and H4.V were absent, chromatin organization and accessibility of VSG-encoding sequences in expression sites were increased compared to wild-type parasite, suggesting that the process is complex and cannot be solely explained by chromosomal reorganization alone [88]. These results not only highlight the importance of histone marks on chromosomal reorganization but also remind us that chromosomal reorganization on its own may not explain the full extent of transcription regulation.

A more recent paper published by the Siegel group followed up on the role of the VSG genes contacts in the three-dimensional genomic space. They discovered that the highly expressed VSG genes were in close contact with an array of splice leader RNA (SL-RNA) genes, genes responsible for *trans*-splicing RNA [90]. Using Hi-C, they also established that the highly expressed VSG genes were in proximity of SL-RNA genes despite their interchromosomal nature, more so than any intrachromosomal loci. They determined this characteristic for 2 different VSG genes, VSG-2 and VSG-13, which were highly expressed in 2 different isogenic lines. They also showed that during a different stage, another highly expressed surface antigen called procyclin genes clustered with the VSG genes in a VEX2 knockdown line, demonstrating the consistency of this interaction between SL-RNA genes and the surface antigens [90]. Interestingly, inhibition of splicing via sinefungin resulted in a loss of interaction between VSG genes and the SL-RNA genes. This highlights a completely different function of nuclear architecture, where proximity between genes may highlight changes not only in transcription but also in RNA processing [90], further bolstering the role three-dimensional architecture may play in infectious diseases.

An additional work published very recently also highlight the role of 3D genome organization and gene expression in the parasite *Trichomonas vaginalis*. *T*. vaginalis is another protozoan parasite responsible for trichomoniasis, a sexually transmitted infection and the most common protozoan infection in industrialized countries with over 160 million of new infection per year worldwide [91]. While generally asymptomatic, *T*. *vaginalis* infection can cause vaginitis, urethritis, and prostatitis. If untreated, the infection can increase the risk of complication during pregnancy, acquiring and transmitting other sexually transmitted disease including human immunodeficiency virus (HIV) and the possibility of developing cervical or prostate cancer. As of today, *T*. *vaginalis* genome is the largest protozoan parasite genome ever sequenced. Only half of the 46,000 protein-coding genes seems to be expressed, but we still lack a clear understanding of the molecular mechanism driving the gene expression. In this new paper, Lizarraga and colleagues found not only the presence of DNA methylation [N6-methyladenine (6mA)] in *T*. *vaginalis* genome but their data also suggest that 6mA is associated with transcriptional repression [92]. Using 3C experiments, they also demonstrated that 6mA is associated with chromatin loop formation, modulation of gene expression, and the 3D genome architecture in this deep-branching eukaryote [92].

### Pathogen effects on host nuclear architecture

Thus far, the focus has been placed on the pathogen’s nuclear architecture, especially chromosomal reorganization of protozoans during their life cycle progression. However, there is an increasing wealth of literature on the effects of infectious disease agents on the nuclear architecture of the host cell, especially in viruses such as HIV. As part of their replication process, viruses must integrate parts of their genome into the host genome to transcribe and translate proteins essential for proliferation. During this process, data have now showed that the viruses can alter the host genomic architecture in favor of expressing or silencing certain genes. Some examples include altering epigenetic landscapes, recruiting transcription factors, and utilizing DNA repair mechanisms.

One strong example is the HIV. Retroviruses like HIV integrate their genetic material into the host genome and utilize the host transcriptional machinery. Interestingly, integration of the genetic material into the host is not random. Roughly 70% of HIV provirus is integrated into introns of actively transcribed genes, which tend to cluster around nuclear pore complexes, highlighting the chromatin structure’s role in HIV integration [93]. HIV also utilizes local machinery, such as the Ini-1/SNF5, an ATP-dependent chromatin remodeling factor, which displaces nucleosomes to increase DNA accessibility for HIV integration [94]. Further evidence suggests that HIV integration occurs more heavily at the outer regions of the nucleus and avoids central regions, highlighting yet again that host nuclear architecture may play a significant role in viral integration [93,95].

**Fig 4:**
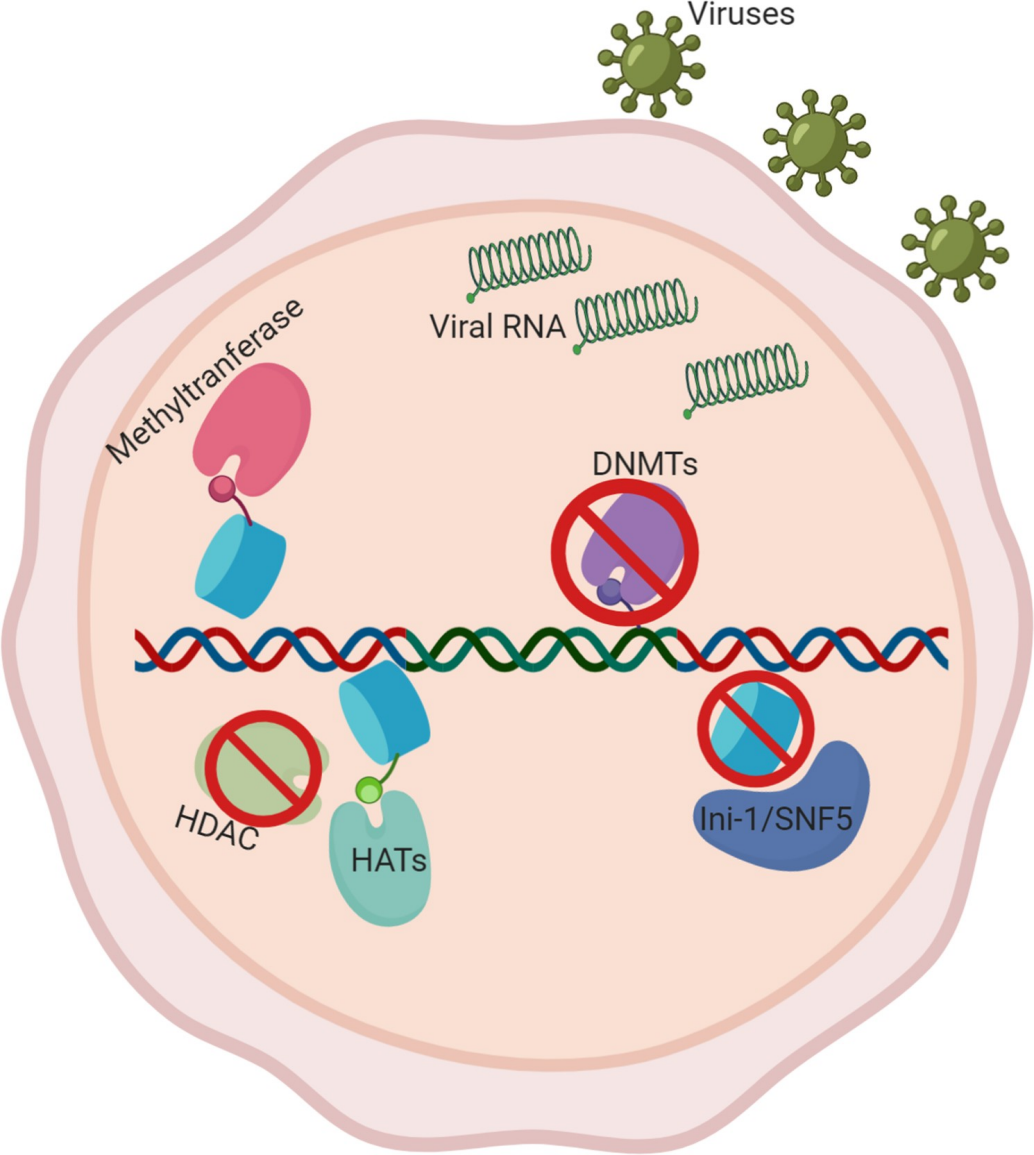
Host nuclear architecture involved in proviral infection. Viral RNA enters the nucleus and integrates into the host genome, typically in the intron of actively transcribed genes. Recruitment of methyltransferases and HATs on host’s nuclear architecture promotes viral proliferation, while inhibition of DNMTs, HDACs, and histone marks creates a similar effect. Figure created with biorender.com. DNMT, DNA methyltransferase; HAT, histone acetyltransferase; HDAC, histone deacetylase.

On top of integration, HIV also utilizes host nuclear architecture for transcription. One such example is histone modifications. Histone acetyltransferases (HATs), such as CBP/p300, P/CAF, and GCN5 acetylate histones, which typically increase nucleosome accessibility, allowing for HIV transcription [96]. Histone deacetylases (HDACs), on the other hand, decrease nucleosome accessibility and are known to prevent HIV transcription, leading to a possible therapeutic target [97]. One such target is BRD4, a protein that recognizes acetylated histones and transcription factors. BRD4 competes with other transcription trans-activator Tat, inhibiting kinase activities of the HIV transcription initiation complex and suppressing the viral machinery [98]. Histone methylation is also known to play a role in HIV transcription, such as the case of SET1 and Smyd2, which methylate histones and promote proviral transcription [99]. Another example of host nuclear architecture playing a role in proviral transcription can be seen through the addition and removal of nucleosomes. For example, the FACT complex (facilitates chromatin transcription), is known to assemble nucleosomes along proviral DNA, silencing HIV transcription (Fig 4) [100]. The above evidence suggests the host nuclear architecture can have a significant effect on the infectious disease progression.

More recent reports offer differing insight into aspects that may also affect viral infection success rates [100–102]. In HCT116 epithelial cell line, commonly used for human colon cancer studies, data suggest that DNA methyltransferase enzymes (DNMTs) and HDACs may have an effect on antiviral responses [101]. Both of these enzymes have a role in epigenetics and change in chromatin structures. DNMTs are responsible for DNA methylation, which typically suppress transcription. By inhibiting DNMTs and HDACs, Hennessey and colleagues found that transcription of Toll-like receptor 3 (TLR3), a pattern recognition protein for the innate immune system, was significantly down-regulated [101]. These data not only highlight once again the role of epigenetics in viral response but also provide a potential avenue to combat viral infections through epigenetic manipulations.

HIV has been the primary focus for its effects on host nuclear architecture, but recent studies have started to highlight other factors of nuclear architecture that can be affected by other viral agents, such as the case of the Epstein Barr Virus (EBV) [102]. EBV, also known as human herpesvirus 4, is the causative agent of infectious mononucleosis and is one of the most common human viruses [103]. Jiang and colleagues studied the effects of an EBV infection in B cells that are transformed to lymphoblastoid cell lines (LCLs) as a result of the infection, via EBV nuclear antigens (EBNAs) [102]. Deletion of specific EBNAs, such as EBNA2 sites, would significantly reduce gene expression required for the establishment and proliferation of LCLs, and hence, the survivability of EBVs [102]. Additionally, inactivation of EBNAs such as EBNA2 altered the structure of the LCL chromatin, reducing the chromatin looping between EBV super enhancers (hotspots of EBNA targets) and MYC transcription start sites, a critical family of genes required for the proliferation of LCLs and EBVs [102]. These results highlight another drastic altercation of host nuclear architecture that viral agents can pose on their host’s chromatin conformation at local levels.

Although there are no reports of dynamic viral effects on host chromosome reorganization to the extent seen in *P*. *falciparum*, Deng and colleagues reported that herpes simplex virus-1 (HSV-1) can affect the telomere structures of their host [104]. HSV-1, the causative agent of oral herpes, is estimated to affect about 3.7 billion people under age 50 globally [105]. Deng and colleagues showed that human diploid fibroblasts infected by HSV-1 had a significant amount of chromosomal structural aberration and DNA damage at the telomere ends [104]. HSV-1 infection not only significantly induced transcription of noncoding RNA TERRA, known to play a role in telomere regulation, but also decreased the distribution of histone H3 and nucleosomes. Furthermore, HSV-1 triggered a significant loss of core shelterin proteins TRF1 and TRF2 that are known to bind to telomere repeat DNA. Altogether, these data suggest that nucleosomes, along with shelterin, are depleted from telomeres during HSV-1 infection to promote viral replication [104]. A better understanding of the molecular mechanisms leading to chromosome reorganization by these pathogens could lead to the development of novel therapeutic strategies against many infectious diseases.

While viruses are most famous for their direct interactions with host nuclear architecture, recent literature has started to highlight how eukaryotic pathogens, such as apicomplexans or trypanosomes, can also disrupt the host nuclear architecture. *T*. *gondii*, like many other infectious disease agents, secrete proteins into their host cell. One such protein named *Toxoplasma* E2F4-associated EZH2-inducing Gene Regulator (TEEGR) is excreted and exported to the host nucleus to alter the host’s transcriptional response [106]. TEEGR has been showed to bind to 2 transcription factors, E2F3 and E2F4, and their binding partners to create an approximately 500 kDa protein complex [106] and significantly alter the host transcription, including down-regulation of NF-κB leading to a significant decreased in the host immune response [106]. Mice infected with parasite deficient in TEEGR were protected compared to mice infected with wild-type counterparts, suggesting that TEEGR alters host nuclear architecture and transcription and plays a vital role in pathogen survivability. In addition to TEEGR, *T*. *gondii*, inhibitor of STAT1 transcription (TgIST), has also been reported as a *T*. *gondii-*secreted protein that decreases STAT1 expression [107,108] known to be involved in host inflammatory responses [107,108]. TgIST-deficient parasites were rapidly cleared by the hosts, demonstrating once again that an infectious agent can affect the host nuclear architecture and mechanisms regulating transcription [107,108].

Similar to *T*. *gondii*, *Trypanosoma cruzi*, a parasite responsible for Chagas disease, can also secrete proteins into the host nucleus and affect the nuclear architecture. One such protein is the *T*. *cruzi* High Mobility Group B (TcHMGB) protein that affect nuclear architecture by interacting with nucleosomes, transcription factors, and histones [109]. HMGB proteins are found commonly in a wide range of organisms, including humans, and play a crucial role in lymphocyte development and immune response [109]. TcHMGB, when acetylated, relocates from the pathogen’s nucleus to the cytoplasm and is consequently secreted to the host cell [110]. Genetic modification of HMGB in *T*. *cruzi* increases the host immune response, suggesting that this protein plays a significant role in pathogen survival [110]. A more recent study demonstrates that overexpression of TcHMGB affects parasite’s nuclear architecture, transcription initiation, and the size of the parasite nucleolus, and alters the heterochromatin and euchromatin ratios [111]. It is highly possible that secreted TcHMGB may alter the host nuclear architecture in a similar manner, but further studies will be required to determine the exact molecular effects of TcHMGB on the host nucleus.

*Leishmania donovoni*, another protozoan parasite causing leishmaniasis, can infect and replicate within human phagolysosomes of macrophages and affect directly the host immune system. Recently, Roy and colleagues demonstrated that macrophages infected by *L*. *donovani* [112] exhibit a down-regulation of host defense transcripts, such as DEFA, MPO, and PTEN [112]. They further determined that this down-regulation was dependent on HDACs and could be reversed by HDAC inhibition, both genetically (through siRNA) and pharmacologically (by sodium butyrate) leading to decreased propagation of *L*. *donovani* [112,113]. Although the down-regulation of host defense genes is most likely due to molecular components secreted by *L*. *donovani* (either protein or RNA), the identification of these factors and their direct effect on chromosomal reorganization will need to be further investigated.

## Conclusions

In this review, we covered our current knowledge regarding chromosomal reorganization in infectious disease. A large body of recently published work focused on chromosome reorganization in *Plasmodium* and its potential role in regulating transcription and stage progression. In other organisms such as *T*. *gondii* and *T*. *brucei*, the relationship between transcription and chromosomal reorganization is not as clear. Despite the evolutionary similarity between these organisms, the divergence of the role of chromosomal organization raises questions about how and why these divergences occurred.

This review also includes our current knowledge of pathogens altering the host cell nuclear architecture, including viruses that are known to manipulate, and exploiting the host nuclear architecture for their own proliferation. Data have demonstrated that viral agents can alter the host epigenomes not only by affecting level of histone methylation and acetylation to increase or decrease DNA accessibility but also by recruiting the transcriptional machinery to favor their viral replication. Many of these pathways have been sought out as novel therapeutic targets. Although there is only select evidence of virus affecting chromosomal reorganization, it will be interesting to see if some of these viruses can affect this process for their benefit in a more dynamic matter. Other pathogens such as apicomplexans and trypanosomes can also affect host nuclear architecture in their favor. Most evidence currently points to parasites attempting to alter the host immune responses to favor their proliferation. In *T*. *cruzi*, molecular components such as TcHMGB protein can directly or indirectly alter not only the parasite but also the host chromosomal organization.

There is much to be learned about the interactions of pathogens and nuclear architecture, both in the infectious disease agent itself as well as in the host cell. While challenging to investigate in a more systematic manner, recent advances in technologies and access to novel experimental approaches such as Hi-C and Chip-seq allowed mold-breaking discovery in the role of chromatin structure in infectious disease progression. It is most likely that access to more advanced technologies and improved resolution including simultaneous profiling of 3D genome structure, epigenetics, and change in gene expression in both infectious agents and host cells at single-cell resolution will soon revolutionize the field and reveal specific chromatin conformation maps suggesting pervasive interactions between epigenetic processes and gene expression in most infectious diseases.
